# The Intestinal Flora Is Required to Support Antibody Responses to Systemic Immunization in Infant and Germ Free Mice

**DOI:** 10.1371/journal.pone.0027662

**Published:** 2011-11-17

**Authors:** Esi S. Lamousé-Smith, Alice Tzeng, Michael N. Starnbach

**Affiliations:** 1 Division of Gastroenterology and Nutrition, Children's Hospital Boston, Boston, Massachusetts, United States of America; 2 Department of Microbiology and Immunobiology, Harvard Medical School, Boston, Massachusetts, United States of America; Charité-University Medicine Berlin, Germany

## Abstract

The presence of a complex and diverse intestinal flora is functionally important for regulating intestinal mucosal immune responses. However, the extent to which a balanced intestinal flora regulates systemic immune responses is still being defined. In order to specifically examine whether the acquisition of a less complex flora influences responses to immunization in the pre-weaning stages of life, we utilize a model in which infant mice acquire an intestinal flora from their mothers that has been altered by broad-spectrum antibiotics. In this model, pregnant dams are treated with a cocktail of antibiotics that alters both the density and microbial diversity of the intestinal flora. After challenge with a subcutaneous immunization, the antibiotic altered flora infant mice have lower antigen specific antibody titers compared to control age-matched mice. In a second model, we examined germ free (GF) mice to analyze how the complete lack of flora influences the ability to mount normal antibody responses following subcutaneous immunization. GF mice do not respond well to immunization and introduction of a normal flora into GF mice restores the capacity of these mice to respond. These results indicate that a gastrointestinal flora reduced in density and complexity at critical time points during development adversely impacts immune responses to systemic antigens.

## Introduction

The gastrointestinal tract (GIT), in terms of mass and cellularity, is the largest immune organ in the body. Complex symbiotic interactions have evolved between the intestinal flora and mammalian host to nurture the development and function of the immune system. In mice that develop without an intestinal flora (germ free mice) intestinal microbial colonization stimulates the maturation of mucosal (i.e. peyers patches, lamina propria) and systemic (i.e. spleen, thymus, lymph nodes) lymphoid tissues [1], [2]. Within these tissues, the stable differentiation, expansion, and maintenance of cell populations with ascribed adaptive immune function have now been shown to be dependent upon intestinal microbial colonization. These populations include: CD4^+^FoxP3^+^ regulatory T (Treg) cells, Th17 cells, and γδ^+^T cells [3], [4], [5], [6], [7]. Strikingly, deficiencies of these cell populations, as noted in germ free mice or as induced by treatment with antibiotics to alter the intestinal flora [8], are also associated with immune dysregulation and the development of allergic and autoimmune diseases (i.e. type 1 diabetes, rheumatoid arthritis, inflammatory bowel disease) in humans and in mouse models [9], [10], [11], [12]. Together, these observations indicate that the intestinal flora has broadly-distributed effects on adaptive immune function and its regulation. The exact mechanisms involved in how members of the intestinal flora mediate these effects are not yet entirely clear.

The period of infancy is important for “setting the stage” for future immune regulation and the intestinal flora may have an important role to play in this. How the GIT flora influences both the development and function of systemic adaptive immunity during this period of development has not been well studied. Unlike adults, in infants the composition of the intestinal flora undergoes dramatic changes during the maturation of the intestine from birth to weaning [13], [14], [15]. The progress of colonization during this time period may be altered or interrupted by external influences such as manner of birth, infection, or exposure to antibiotics. As determined by culture and deep sequencing of the stool flora, antibiotic exposure early in life alters the intestinal flora of infants [14], [16] and reduces its biodiversity often allowing more pathogenic commensal organisms to establish niches thus potentially outcompeting more beneficial commensal organisms [15], [17]. Up until recently, it has been generally accepted that antibiotic use causes only transient changes to the GIT flora and has no significant future consequence, however antibiotics cause dramatic alterations to the GIT flora, both in density and diversity, that does not always return to baseline [18], [19].

Dysbiosis, defined as an imbalanced or aberrant microflora, has likely implications for immune function, since the depletion of commensal species is correlated with the loss of immune cell populations important for regulating immune responses. Therefore, alterations in the intestinal flora during infancy and early childhood may not be benign, particularly since this is a period when first exposures to a barrage of mucosal and systemically delivered antigens is occurring in tandem with both tissue and functional development of the immune system. Clinically, the rising incidence of allergic and autoimmune diseases in infants and children is thought to be partially influenced by intestinal dysbiosis that causes immune system defects [20].

The effects of antibiotic use during pregnancy on both the intestinal microflora and adaptive immune function of infants has not been extensively studied. What are the consequences of altering the intestinal flora on systemic adaptive immune function and at what time periods during infancy and childhood are such functional differences detectable and significant? We sought to determine whether the normal establishment of the GIT flora at birth and during infancy is a requisite for adaptive immune function to a systemic antigen challenge. In the experiments presented here, we utilized a model in which broad-spectrum antibiotics are administered to pregnant mice and demonstrate that the dams and the pups born to these dams both have similar alterations in the composition of their GIT flora. The alterations in gut flora were associated with deficiencies in serum antibody responses in infant mice challenged to a systemic vaccination. Similar deficiencies were also found when GF mice were immunized systemically, and were reversed by allowing the GF mice to acquire a complex intestinal flora.

## Materials and Methods

### Animals

C57BL/6J GF mice were obtained under contract from Taconic (Hudson, NY) at 6–12 weeks of age and housed in the Harvard Digestive Diseases Center Germ Free Core facility. Germ free status was confirmed by weekly stool analysis for aerobic and anaerobic bacteria. Pairs of age matched adult conventional mice C57BL/6J mice were obtained from the Jackson Laboratory (Bar Harbor, ME), and housed in our facility prior to breeding. All experiments and protocols were approved by Harvard Medical School's Institutional Animal Care and Use Committee.

### Bacterial Colonization of Germ Free Mice

In some experiments, we intentionally colonized GF mice. One day after arrival into our pathogen-free facility, we mixed dirty bedding from conventional strain matched mice into the bedding of the GF mice and also rubbed dirty bedding onto their fur. Aerobic and anaerobic stool cultures were performed weekly to monitor colonization.

### Antibiotic Administration to alter Stool Flora

Ampicillin (AAP Pharmaceuticals), Streptomycin (X-gen Pharmaceuticals), and Clindamycin (Cleocin Pediatric, Pharmacia and Upjohn Co.) were mixed into sterile drinking water at a final concentration of 1 mg/ml. Mice were allowed to drink the water *ad libitum* for the duration of the experiments. Antibiotic-containing water was given to pregnant dams at least 3–5 days prior to expected birth of a litter. Water was replaced and/or refreshed every 3 days for the duration of the experiment (i.e. until pups were sacrificed). At sacrifice, stool or cecal contents were collected weighed, and cultured or snap frozen for storage at −80°C prior to DNA extraction.

### Stool and Cecal DNA extraction and quantitative PCR

DNA was extracted from snap frozen stool or cecal stool using the QIAamp Stool DNA mini-kit (Qiagen) according to manufacturer's instructions. Ceca were homogenized in 2ml sterile PBS and DNA isolated as above. The abundance of total and specific intestinal bacterial groups (Bacteroidetes, Enterobactericeae, Firmicutes) was measured by qPCR using universal and group-specific 16S rRNA gene primers (Table S1) and the QuantiTect SYBR Green PCR kit (Qiagen). Bacterial DNA purified from the stool and cecal contents was used to approximate the bacterial community of the large intestine. qPCR was performed on an ABI Prism 7000 Sequence Detection System (Applied Biosystems). Bacterial DNA was quantified using standard curves constructed with reference bacteria specific for each bacterial group analyzed.

### Immunizations

A suspension of ovalbumin (Grade III, Sigma) and complete Freund's adjuvant (MP Biomed) was prepared by combining equal volumes of ovalbumin and CFA. Mice were immunized by subcutaneous injection of the suspension into the internal flank. The final injection dose of ovalbumin was 250 micrograms for adult mice (≥21 days of age) and 100 micrograms for infant mice (≤14 days of age).

### ELISA

For serum collection, mice were sacrificed 10 days after immunization. Serum was extracted from blood collected by cardiac puncture and stored at −20°C until use in sandwich ELISA to detect Ova specific antibodies [21]. Antibodies (unlabeled and HRP-conjugated goat anti-mouse IgG; purified mouse IgG) were purchased from Southern Biotech. Ovalbumin (Grade 6, Sigma) was used to coat the ELISA plates. For total serum IgG, the ELISA plates were coated with anti-mouse IgG. After blocking and washing the plates, the serum was diluted for detection. After 2–4 hours at room temperature, the plates were washed and HRP labeled anti-mouse IgG was added for one hour. Plates were developed by adding 100 microliters of TMB (Sigma Aldrich) and then stopped with 50 microliters of 2N H_2_SO_4_. The absorbance was read at 450 nm and the results are expressed at one dilution as ng/ml based on standard curve.

## Results

### Antibiotics cause reproducible shifts in the intestinal microflora of adult and infant mice

Recent studies have shown that antibiotics significantly alter the complexity and diversity of the intestinal flora, at both phylum and species levels [8], [9], [14], [18], [22]. The consequence of antibiotic-induced alterations of the GIT flora on host physiology is presently generating intense interest. Even short courses of antibiotics alter the GIT flora and epidemiologic studies in human infants and children have suggested that antibiotic exposures early in life may be a significant risk factor in the development of future allergic and autoimmune diseases [11], [23], [24], [25]. Therefore the timing of exposure to flora-altering agents (antibiotics, infection, diet) is important to understanding how and why certain autoimmune and allergic diseases develop, particularly since the interactions between flora and host are occurring on the background of an immune system that is still maturing during the first days, months, and years of life. Here we used mouse models to explore whether alterations in the composition of intestinal flora, or lack intestinal flora altogether, influence the immune responses to systemic vaccination.

**Figure 1:**
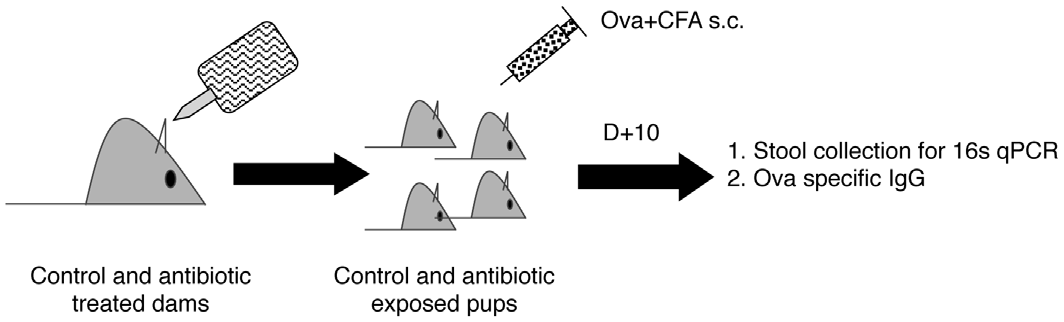
Generation of antibiotic exposed infant mice. Pregnant dams were treated with antibiotics in the drinking water during the last 3–5 days of gestation. Antibiotics were supplied throughout the duration of the experimental period. Infant mice born to control (no antibiotics) or treated (with antibiotics) mice were immunized by subcutaneous injection at ages specified. Ten days following immunization, serum and stool were collected from immunized mice for analysis.

To alter the GIT flora, we selected a combination of ampicillin, clindamycin, and streptomycin for their broad-spectrum activity against bacteria represented within the intestinal flora of mice and humans. We have found that this combination appreciably, but not completely, reduces gram positive and negative microbes in the GIT flora of adult mice. To alter the GIT flora of infant mice, pregnant dams were treated with this cocktail in their drinking water for at least 3–5 days prior to birth of a litter and throughout the duration of the suckling/pre-weaning period (Figure 1). In this model, infant mice are born to a mother with an altered intestinal (and likely also vaginal) flora and are continuously exposed to this altered flora as well as minute quantities of antibiotics during nursing. There were no appreciable differences in the size of litters or mice between treated (Abx) and untreated control (Ctrl) groups and neither did any adult mice treated with antibiotics demonstrate dehydration or weight loss.

To detect compositional changes in the intestinal microbial flora mediated by antibiotic treatment we performed qPCR with universal bacterial and group-specific primers targeting the 16S rRNA genes of Bacteroidetes, Enterobacteriaceae, and Firmicutes. We used qPCR as opposed to stool cultures, since culture may under or overestimate bacterial density due to variation in growth patterns under aerobic and anaerobic conditions on non-selective media. Taken together the phyla we examined represent the majority of bacterial species in the murine GIT flora [18], [26]. The primers we employed have been shown to identify major changes in total bacteria and the major bacterial subgroups of the mouse GIT consortium [27]. While mass sequencing of DNA from the stool provides more specific information about the flora down to the species level [8], [18], qPCR with phyla-specific primers is a relatively fast and inexpensive alternative for analyzing multiple samples and cohorts of mice [28].

**Figure 2:**
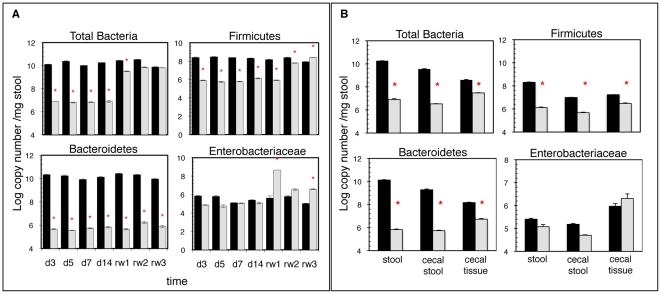
Antibiotic treatment of mice reduces the density and diversity of intestinal microbial flora. A) Fecal microbiota composition is altered by antibiotic treatment and partially recovers 3 weeks after antibiotic withdrawal. Total bacterial genomic DNA was isolated from the stool of adult control (black bars) and antibiotic treated (grey bars) mice at specific time points during and after antibiotic treatment, and the abundance of bacterial groups was detected by qPCR. Fecal pellets were collected on days 3, 5, 7, 14 and 1, 2, and 3 weeks after discontinuing antibiotics (rw1, rw2, rw3). N = 3 mice per group per time point. The data is representative of 3 independent experiments. B) Fecal stool pellets are a proxy for cecal stool. DNA was isolated from fecal pellets, cecal tissue, and cecal stool from adult control and mice treated with antibiotics for 14 days and analyzed by qPCR for total bacteria, Firmicutes, Bacteroidetes, and Enterobacteriaceae phylotypes. N = 2 control and n = 3 Abx treated mice. The data is representative of 3 independent experiments. *P<0.01 compared to untreated controls (determined by Student's t test).

We found that our antibiotic cocktail effectively and reproducibly reduced the total bacterial load in the stool by 2–3 logs as compared to control mice (Figure 2A). Specifically, Firmicutes and Bacteroidetes were significantly depleted. While the populations of Firmicutes recovered after antibiotic withdrawal, the populations of Bacteroidetes remained depleted in adult mice up to three weeks after the discontinuation of antibiotics (Figure 2A), indicating that even relatively short course of antibiotics have a prolonged impact on the intestinal tract flora. By contrast, the Enterobacteriaceae dominated within the flora of antibiotic treated mice and eventually stabilized slightly above or at control levels.

Antibiotic treatment of adult mice induced similar effects on the quantities of total and specific bacterial groups in murine cecal contents and tissues (Figure 2B). Therefore, analysis of stool pellets is an appropriate proxy for the composition of the cecal stool consortium. Our results correspond with other published reports where similar shifts in the consortium of intestinal microbial flora were identified by deep sequencing of the intestinal flora following antibiotic treatment of mice [8], [18], [29], [30]. Quantification of bacteria in the cecal tissue, which represent mucosal adherent species, revealed a similar trend: Bacteroidetes,

Firmicutes, and total bacteria manifestly diminished by the end of the two-week regimen, while Enterobacteriaceae expanded by day 14. The cecal tissue-resident bacteria appeared less sensitive to antibiotic effects in general.

**Figure 3:**
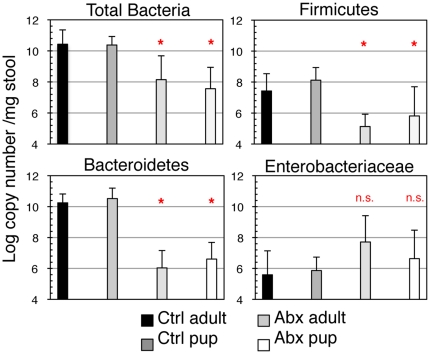
Antibiotic treatment of the mouse dam alters bacterial colonization of her litter. Fecal pellets were collected from dams and from the cecal contents of their pups. Data are pooled from 3 individual experiments: n = 3 dams, n = 17 ctrl pups and n = 12 abx pups. Cecal stool contents were collected from male and female pups at ages 18–21. *P≤0.05 comparing antibiotic treated adult with control adult and antibiotic exposed pups with control pups, respectively.

In examining the intestinal flora of infant pups born to and nursing from female mice treated with antibiotics, we found that their flora was affected in a manner similar to adult mice (Figure 3). To analyze the total bacterial load and representation of phyla in infant mice (7–14 days old), we pooled the cecal contents of infant mice since the quantity of stool pellets that can be collected from infant mice was insufficient for DNA extraction and qPCR analysis. Pups were not weaned from mothers prior to stool analysis so as to limit the influence of shifts in nutritional intake on the intestinal flora. Infant mice born to and exposed to antibiotics from their mothers exhibited a significant reduction in total bacteria as compared to Ctrl pups, and the changes in Firmicutes, Bacteroidetes, and Enterobacteriaceae populations mirrored that of their mothers. Treatment of pregnant or breastfeeding mothers with broad-spectrum antibiotics has been associated with higher-than-normal densities of Enterobacteriaceae in their infants [29], [31]. We have replicated this circumstance in our mouse model and demonstrate that at the phyla-level, antibiotic altered flora is transmitted to infant pups from their antibiotic treated dams.

As others have shown [32], [33], we also observed that antibiotic-induced changes of the flora alter host cell gene expression, and found significantly reduced expression of cryptidins, RegIIIγ, and RelMβ in adult mice (data not shown). In addition, we found reduced expression of Nod2 and Rip2, but not MyD88 expression and these data are consistent with the previously reported dependence of Nod2 expression on the presence of commensal flora [34].

### Young mice with altered GIT flora have lower antigen specific serum antibodies following immunization

**Figure 4:**
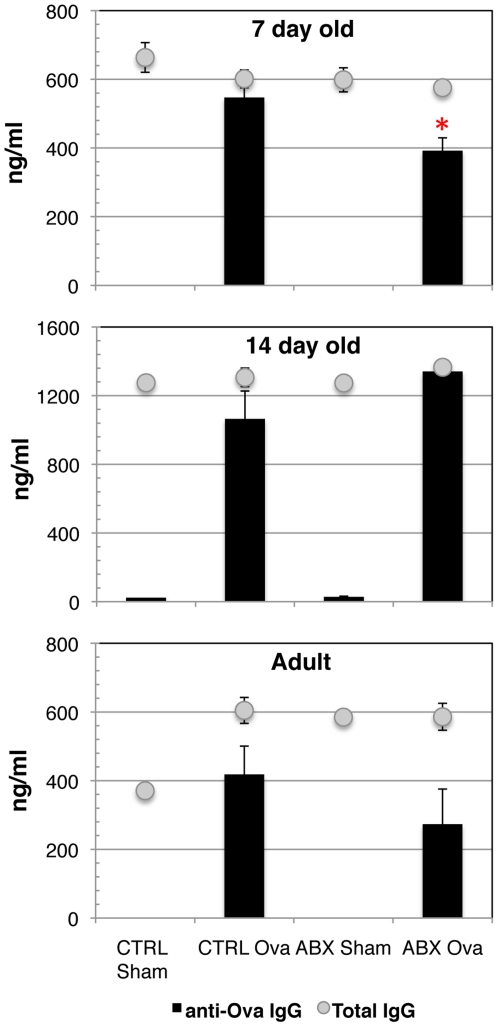
Ova-specific IgG titers in antibiotic treated and non-antibiotic treated control mice following immunization. Ten days after subcutaneous immunization with ovalbumin and CFA, serum was collected from mice immunized as adults (>6 weeks old), at 14 days of age, or at 7 days of age. Ova-specific IgG titers were detected by ELISA. Total serum IgG was equivalent among groups. Representative data of three experiments are shown (3–7 mice per group). *p<0.05, unpaired student t-test.

To address whether changes in the composition of the gastrointestinal tract flora would result in a measurable reduction in systemic immune responses, we vaccinated Abx treated and Ctrl mice by subcutaneous immunization with ovalbumin (Ova) and complete Freund's adjuvant (CFA). Ten days after immunization, sera from the mice were analyzed for the presence of ova-specific antibodies. Pups remained with their mothers throughout the duration of the experiment and therefore were continuously exposed to antibiotics until serum was collected for analysis. We found no difference in the ova-specific serum antibody response when Ctrl and Abx treated mice were immunized at 14 days of age or at >6 weeks of age (Figure 4). We surmised that there might be a developmental window, earlier in life, during which antigen specific responses would be most affected by perturbations in intestinal flora. Therefore, we analyzed ova-specific antibody titers from the serum of Abx-exposed and Ctrl mice immunized at 7 days of age. As shown in Figure 4, control/non-antibiotic exposed mice had higher titers of ovalbumin specific IgG in their serum as compared to those mice that were exposed to antibiotics. By day of life 14, the ability of Abx exposed mice to respond to systemic immunization returned to reach levels of age matched controls. Total serum IgG was not affected by antibiotic treatment at any age. This indicates that the lack of response to ova in 7 day old infant mice did not result from an overall deficiency in the capacity of B cells to produce IgG. Other factors required to generate antigen specific antibody responses may thus be compromised when the intestinal flora is altered at birth.

### Germ free mice have deficient serum antibody responses following systemic immunization

**Figure 5:**
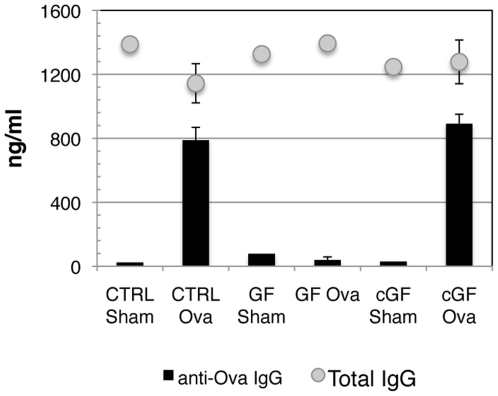
Antibody responses following systemic immunization of GF mice and cGF mice. Twelve week old control, GF, and GF mice colonized with flora (cGF) derived from strain matched mice were immunized with PBS + CFA (Sham) or with Ova+CFA (Ova). Ten days later serum was collected and analyzed for ova-specific IgG (A) and total serum IgG (B). In the representative experiment shown, serum titers are from one sham-immunized and 3 ova-immunized mice. cGF mice were allowed to rest for four weeks following colonization with flora.

Infant mice (and humans) are born with a sterile gastrointestinal mucosa that is rapidly colonized within the first week of life. GF mice, born and maintained with a gastrointestinal tract devoid of microflora, demonstrate deficiencies in the development of immune tissues and immune cells. Their responsiveness to primary systemic immune challenge has not been as extensively examined using the approach of subcutaneous immunization. To examine whether a complete lack of flora results in an inability to mount an antibody response following systemic vaccination, we immunized 12-week old GF and conventional Ctrl mice subcutaneously with Ova and CFA. We consistently observed that GF mice had ova specific antibody titers significantly lower than Ctrl mice (Figure 5). GF mice had profoundly deficient ova-specific antibody responses following vaccination at all ages examined (ages 3–12 weeks), indicating that without flora, developmental age alone could not overcome the deficiency in humoral responses in GF mice. The differences we observed were consistent, whether we measured ova-specific antibody subtype IgG2b or IgG1, corresponding to Th1 and Th2 serum responses respectively (data not shown). It has been previously and recently shown that GF mice have equivalent Ig bearing cells in the spleen [35], [36], [37] and consistent with that, we found that total serum IgG (Figure 5) was fairly equivalent in Ctrl and GF mice. This indicated that the lack of response to ova did not result from an overall deficiency in the ability to produce serum IgG and suggests that other factors required to help antigen specific antibody responses may be deficient in the absence of intestinal flora.

### Germ free mice can respond to systemic vaccination following colonization with a mixed flora

To determine whether the capacity to respond to systemic immunization recovers when GF mice acquire flora, we colonized GF mice with stool from strain matched conventionally-raised SPF control mice. By the end of one week, aerobic and anaerobic stool cultures from the formerly GF mice (referred to as conventionalized germ free mice; cGF) grew out mixed colonies. Two weeks after conventionalization, we estimated the density of colonization to be similar between Ctrl and cGF mice (data not shown). We then examined the serum antibody responses stimulated by vaccination in GF mice, Ctrl mice, and cGF mice. At early time points after conventionalization we observed only partial capacity of animals to mount antigen specific antibody responses (data not shown). However, if mice were allowed four weeks or more to conventionalize, every member of the experimental group mounted an antigen specific antibody response (Figure 5). Therefore, it appears that there is a minimum timeframe required for the development of factors, stimulated by flora, necessary for the response to systemic vaccination.

## Discussion

We were interested in addressing the global question of whether the intestinal flora is required to support systemic immune responses during early periods of growth and development. We thus focused our analysis on antibody responses during the period of infancy to weaning by using antibiotics to alter the intestinal flora. To date, most studies have analyzed how antibiotics alter the GIT flora, immune cell populations, or epithelial gene expression. Fewer have specifically interrogated how altering the intestinal flora influences systemic immune function in response to immunization or infection. Most studies that have analyzed immune function in the setting of an altered GIT flora have focused on the response to antigens introduced into the intestinal lumen (oral tolerance). These studies have determined a relationship between oral tolerance and GIT microbial composition in both adult and infant mice. The mechanisms mediated by the flora for oral tolerance induction are not yet clear, but are probably mediated by shifts in TLR signaling, Th1/Th2 balance, and/or due to alterations of antigen presenting cells in the intestinal tract [38], [39], [40]. Recently the response of adult mice to influenza infection of the respiratory tract following 4 weeks of antibiotic treatment was interrogated [41]. Those adult mice demonstrated deficient T and B cell responses to influenza but not to other viruses or to a subcutaneous injection with ovalbumin. We also found that antibiotic treatment does not influence the antigen specific antibody response following subcutaneous immunization with ovalbumin. However, our focus on the period from birth to weaning indicates that there is a developmental window during which time alterations in flora can influence systemic immune responses. In our experiments acquisition of an altered flora at birth appears to affect primary antibody responses following a systemic immunization that recovers as the infant ages. The results of systemic immunization are even more pronounced when there is a complete lack of intestinal flora as confirmed in germ free mice.

Altering the maternal GIT flora with antibiotics confers an altered flora to their pups at birth, as has been demonstrated in a few studies in rodents that also focused on this period [31], [42]. Our analysis does not provide the sensitivity to determine which eliminated members of the flora correlate with the observed immune deficiencies. This will require sequence analysis of the intestinal flora to obtain resolution of the bacterial species affected by the antibiotics used to alter the intestinal flora. This information would provide one piece of the mechanistic puzzle to explain why antigen specific antibody deficiencies are detected when the flora is either absent or altered. B cell subsets appear to be preserved in germ free mice [43], but the presence of the intestinal flora clearly enhances the capacity of B cells to function properly by responding to T cell dependent antigens. Based upon our data, this also appears to be the case during infancy when the intestinal flora is altered by antibiotics.

In general, infants are highly susceptible to pathogens. This may be due to low numbers or poor functionality of innate and cellular immune effectors, leading to overwhelmingly high titers of invading pathogens [44], [45], [46], [47]. We found that control infant mice can effectively respond to a peripheral immunization. However, in infant mice with an altered flora, the antigen specific antibody response is blunted despite normal levels of serum IgG. Although we have not yet clearly defined the mechanisms involved, deficiencies in immune effectors required for T cell dependent antibody responses (CD4 Th cell and dendritic cell subsets) that rely on stimulation by the normal transitions in the acquisition of a GIT flora are likely to play a role [13]. Adaptive B2 B cells rely on a commensal flora for normal gene expression and function [43] and a reduced population of Th17 cells caused by the lack of (or altered) flora modulates the development of autoimmune arthritis, asthma, and Type 1 diabetes in genetically susceptible GF and antibiotic treated mice [10], [11], [48]. Thus, perturbing the relative abundances of gut microbial phyla or specific commensal species is sufficient to modify mucosal and systemic cellular immune populations required for a properly balanced response to pathogens and other systemic immune challenges. Although not yet explored, the potential for immune dysfunction due to changes in the abundance of these effectors may be greater during infancy when the immune system is still under development.

Another possible mechanism for the immune deficits we have observed in both antibiotic treated infant and GF mice could be attributed to disorganized spleen or lymph node architecture. We have noted subtle changes in the splenic architecture of antibiotic treated infant mice, and it is clear that immune tissue organization and cellular development are disrupted when the intestinal flora is limited or corrupted [3], [7], [49], [50], [51]. GF mice have defects in mucosal and systemic lymphoid structures [52] and antibiotic treatment may also impact immune tissue structure and development due to reduced/altered expression of microbial factors that are required to induce tissue organization [53], [54]. Peripheral immune tissues (spleen and draining lymph nodes) provide a necessary scaffold to facilitate trafficking and accumulation of precursor B cells, T cells, and antigen presenting cells during the initiation of primary immune responses. Therefore, disruption of the organization and architecture of peripheral immune tissues could limit the generation and magnitude of immune responses.

Altogether, our observations indicate that seemingly benign, and even transient, alterations of the GIT flora have far-reaching influences on responses to systemic infection – and the influence of the GIT flora on immune function also corresponds to developmental age. The dam-infant model presented here has potential relevance to the human circumstance, particularly when full- or pre-term infants are born to mothers who receive antibiotics prior to or during delivery. In this setting, it has been shown that the prevalence of colonization with certain bacteria is indeed altered in infants born in these settings [16]. Using these mouse models we can determine the relative influence of intestinal dysbiosis on immune mechanisms that regulate responses to other systemic infections, immunizations, and adjuvants. Defining with greater resolution the members of the gut microbial community that are lost or over-represented following antibiotic treatment will also allow us to test whether reconstitution with these bacteria or with probiotics can also repair the capacity to respond to vaccination.

## Supporting Information

Table S1 **16S rRNA gene universal bacterial and group-specific primers for qPCR.** Primer names, as identified in the original references, are indicated below each group name. Reaction conditions for all primer pairs except the Firmicutes-specific pair (Firm350F/Firm814R): initial 15-min denaturation step at 95°C; 40 cycles at 95°C for 10 s, annealing temperature (AT) for 45 s. Data were acquired in the annealing step at the AT. For Firmicutes-specific qPCR, the following program was run instead: initial 15-min denaturation step at 95°C; 40 cycles at 95°C for 60 s, 56°C for 60 s, 72°C for 30 s. The PCR product sizes are as follows: universal bacteria, 197 bp; Bacteroidetes, 126 bp; Enterobacteriaceae, 355 bp; Firmicutes, 484 bp.
(DOC)
