# Supplementary material for: The Intestinal Flora Is Required to Support Antibody Responses to Systemic Immunization in Infant and Germ Free Mice
Source: PLoS One. 2011 Nov 17;6(11):e27662. doi: 10.1371/journal.pone.0027662 (PMC3219679; doi:10.1371/journal.pone.0027662)
Supplement: Table S1 — 16S rRNA gene universal bacterial and group-specific primers for qPCR. Primer names, as identified in the original references, are indicated below each group name. Reaction conditions for all primer pairs except the Firmicutes-specific pair (Firm350F/Firm814R): initial 15-min denaturation step at 95°C; 40 cycles at 95°C for 10 s, annealing temperature (AT) for 45 s. Data were acquired in the annealing step at the AT. For Firmicutes-specific qPCR, the following program was run instead: initial 15-min denaturation step at 95°C; 40 cycles at 95°C for 60 s, 56°C for 60 s, 72°C for 30 s. The PCR product sizes are as follows: universal bacteria, 197 bp; Bacteroidetes, 126 bp; Enterobacteriaceae, 355 bp; Firmicutes, 484 bp. (DOC) [file pone.0027662.s001.doc]

| **Group** | **Sequence (5’ to 3’)** | **Reference**  **organism** | **AT (**°C) | Reference |
| --- | --- | --- | --- | --- |
| Universal bacteria (Uni340F/Uni514R) | F’: ACTCCTACGGGAGGCAGCAGT  R’: ATTACCGCGGCTGCTGGC | E.coli | 63 | [1] |
| Bacteroidetes (Bact934F/Bact1060R) | F’:GGARCATGTGGTTTAATTCGATGAT  R’: AGCTGACGACAACCATGCAG | *B. fragilis* | 63 | [2] |
| Enterobacteriaceae  (Uni515F/Ent826R) | F’: GTGCCAGCMGCCGCGGTAA R’: GCCTCAAGGGCACAACCTCCAAG | *E. coli* | 67 | [1] |
| Firmicutes  (Firm350F/Firm814R) | F’: GGCAGCAGTRGGGAATCTTC R’: ACACYTAGYACTCATCGTTT | *L. reuteri* | 56 | [3] |

Table References:

1. Barman M, Unold D, Shifley K, Amir E, Hung K, et al. (2008) Enteric salmonellosis disrupts the microbial ecology of the murine gastrointestinal tract. Infect Immun 76: 907-915.

2. Guo X, Xia X, Tang R, Zhou J, Zhao H, et al. (2008) Development of a real-time PCR method for Firmicutes and Bacteroidetes in faeces and its application to quantify intestinal population of obese and lean pigs. Lett Appl Microbiol 47: 367-373.

3. Muhling M, Woolven-Allen J, Murrell JC, Joint I (2008) Improved group-specific PCR primers for denaturing gradient gel electrophoresis analysis of the genetic diversity of complex microbial communities. ISME J 2: 379-392.
